# Migration Strategy and Diet Affect the Metabolism of Passerine Birds During Endurance Flight

**DOI:** 10.1002/ece3.71299

**Published:** 2025-04-15

**Authors:** Susanne Jenni‐Eiermann, Lukas Jenni

**Affiliations:** ^1^ Swiss Ornithological Institute Sempach Switzerland

**Keywords:** day/night migration, diet composition, fat metabolism, migration distance, plasma metabolites, protein metabolism

## Abstract

Bird migration varies greatly in overall distance and length of single flight bouts. Therefore, we expect that metabolic adaptations may also differ widely among migrants. Endurance flight is mainly fuelled by fat and complemented by protein. The proportions of lipids and protein accumulated before flights, and spent during flight, depend on food type. A fruit diet facilitates fattening more than a proteinous arthropod or seed diet. Adaptations to maximize lipid use during flight vary with the length of flight bouts. We expect that the type of diet and migration strategy (length of flight bouts, overall migration distance) affects flight metabolism. On a Swiss Alpine pass, we caught 30 species of nocturnal and diurnal migrant passerines out of natural migratory flight and compared them with conspecifics kept inactive. We examined the effects of migration strategy, primary diet, and body fat stores on plasma concentrations of six metabolites of the fat, protein, and carbohydrate metabolism, used as indicators of relative fuel use. During migratory flight, immediate migration strategy (short hops during day or long bouts during night), general migration strategy (long‐ and short‐distance migration) and diet affected metabolite levels, while fat stores had an additional effect. Triglyceride and free fatty acid levels were high in night‐migrants and frugivores. Uric acid and glucose levels were high in insectivores. Glucose, ß‐hydroxy‐butyrate, and glycerol were less dependent on day/night‐migration or diet. The metabolic profiles indicate that migrant passerines vary in the degree of fat use depending on migration strategy, diet, and current fat stores. Day‐migrating short‐distance migrant insectivores and granivores used protein or glycogen to a higher degree than night migrants. Frugivores maximized fat use. Long‐distance migrants favored fat use even further. Hence, long‐distance night‐migrant frugivores with high fat loads appear best adapted for fat use during migratory flight.

## Graphical abstract

We compared the metabolic profile of 30 bird species caught out of their migratory flight. The degree of fat use depended on migration strategy, diet, and current fat stores. Long‐distance night‐migrant frugivores with high fat loads appear best adapted for fat use during migratory flight, compared to day‐migrants, short‐distance migrants, granivores, and insectivores.

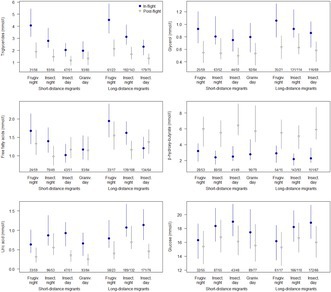

## 1 Introduction

The seasonal migration of birds between the breeding and the non‐breeding grounds varies dramatically in distance and behavior between species and populations. Birds migrate over a large range of distances from a few to 10,000 km or more. Single migration bouts are short hops lasting up to a few hours, which leave sufficient time to feed, or are non‐stop flights covering thousands of km during several days without the opportunity to feed or drink. Moreover, some species migrate exclusively during the day, others only during the night, and some both, day or night. Therefore, it is to be expected that the metabolism, and particularly the kinds of fuel used, vary depending on migration strategy.

Lipids are the main source of energy during migratory flights (e.g., Jenni and Jenni‐Eiermann 1998; Berthold 1996). Correspondingly, long‐distance migrants developed multiple adaptations which maximize lipid use, for example, an increase of fat‐oxidizing enzymes, efficient fatty acid transport in the blood via very low density lipoproteins, an increase in fatty acid transporters across membranes, and a change of muscle fiber composition (Lundgren and Kiessling 1985; McFarlan et al. 2009; Jenni‐Eiermann and Jenni 1992; Lundgren and Kiessling 1988; Guglielmo 2010; Chang et al. 2024). However, endurance flight also needs a certain proportion of protein (e.g., to feed the anaplerotic flux; Dohm et al. 1986; Jenni and Jenni‐Eiermann 1998). The proportions of lipids and protein deposited before flights depend on the type of food. A diet with a high proportion of protein induces lower fat deposits than a low‐protein diet which induces high fat deposits (Rosebrough and McMurtry 1993; Klasing 1998; Kirkpinar and Oguz 1995; Guglielmo et al. 2017; Smith and McWilliams 2010). In turn, diet composition likely affects the proportions of lipids and protein used during migratory flights, as suggested earlier (Jenni‐Eiermann and Jenni 2003).

Diurnal migrants fly shorter distances non‐stop, mainly in the morning, and use the afternoon for foraging (Alerstam 2009; Newton 2008; Dorka 1966), while night‐migrants fly non‐stop for several hours or the entire night (Jenni‐Eiermann et al. 2024; Bruderer and Liechti 1998; Zehnder et al. 2001). It also seems that not all day‐migrants fly non‐stop during the morning hours but intermittently. For instance, we observed many day‐migrating species (e.g., finch species, and very pronounced, in tit species) making a series of short stops on trees and bushes, calling (but not feeding), before they continued their flight (own observations). Hence, migrating during the day, in contrast to night migration, is characterized by daily resting and refueling stopovers and often short stops during flight bouts. Therefore, day‐migrants generally need less fat stores for migration (Newton 2008). Such a flight mode may have consequences for energy metabolism.

We used plasma metabolite concentrations as indicators of relative fuel use, although they cannot generally be equated with metabolite turnover (for more details and discussion see Jenni‐Eiermann and Jenni 1998). The mobilization of extra‐muscular fat stores increases plasma free fatty acids (FFA) and glycerol (GLYC) levels, the products of triglyceride hydrolysation (Jensen et al. 2001). FFA is insoluble in the blood and transported bound to albumin. To increase the capacity of FFA transport, flying and fasting birds additionally resynthesize FFA into triglycerides (TG) which are then released from the liver as very low‐density proteins and carried to the target organs (Jenni‐Eiermann and Jenni 1992). Therefore, plasma levels of FFA, GLYC, and TG in fasting birds during endurance flight are indicators of lipolysis. The ketone body β‐hydroxy‐butyrate (HBA) is synthesized from FFA and is an indicator of the fasting state. In contrast to the above‐mentioned metabolites, its main task is to substitute part of glucose (GLU) in the brain and not to fuel the exercising muscles (Robinson and Williamson 1980; Hue and Taegtmeyer 2009). Uric acid (UA) is the end‐product of protein catabolism and is an indicator of proteolysis (Scanes 2015). Protein is less suited to fuel endurance exercise because its degradation is accompanied by functional losses. However, glucogenic amino acids, after transformation, refill the intermediates of the citric acid cycle enabling FFA oxidation. Therefore, the degradation of protein into amino acids is essential. GLU, a metabolite of carbohydrate metabolism, fuels the onset of flight (Brackenbury and El Sayed 1984; Schwilch et al. 1996).

In this study we analyzed whether migration strategy and diet during energy deposition are related to the metabolic profile of passerine birds during migratory flight. We caught 30 species of nocturnal and diurnal migrants out of their natural migratory flights when they crossed a Swiss Alpine pass. We examined the effects of (a) migration strategy (i.e., whether the species migrates during the day or night and whether it migrates to the Mediterranean basin or to sub‐Saharan Africa), (b) primary diet during refueling (mainly fruit, insects or seeds), and (c) individual body fat stores on plasma concentrations of six metabolites of fat, protein, and carbohydrate metabolism. To evaluate the effect of migratory flight on metabolism, we compared the plasma metabolite concentrations of birds caught directly out of flight (exercising while fasting = in‐flight) with those which were kept inactive for 1–13 h (resting while fasting = post‐flight).

We expected an increase of fat‐ and a reduction of protein‐catabolism in birds which adapt their metabolism to long‐distance flights by the means mentioned above. These would be birds with high body fat stores, species migrating to sub‐Saharan wintering grounds, and birds migrating at night. Further, we expected that a diet poor in protein but rich in glucose or fat would increase fat catabolism and reduce protein catabolism. Hence, the highest dependence on lipids during actual migratory flight is expected to occur in long‐distance night‐migrants feeding on fruit. In contrast, we expected that day‐migrants flying in short hops, which are mainly short‐distance migrating granivores, do not push fat catabolism to a maximum but derive more energy from glycogen than long‐distance night‐migrants.

## 2 Material and Methods

### 2.1 Animals, Migration Strategies and Diet

At the Alpine pass Col de Bretolet (1923 m a.s.l.), Switzerland, free‐living birds were caught when crossing the pass during the autumn migration periods from August to October 1986–1988, 1991, 1992, and 1994. This site in the Alps is a gateway from central to southern Europe and is used by birds migrating from central and northern Europe to southern Europe and trans‐Saharan winter quarters. Night‐migrants were caught in 9 m high mist nets out of their nocturnal migratory flight. Diurnal migrants were caught in these 9 m nets and in 2.5 m high nets. The nets were opened 24 h, if no rain or strong wind; hence, birds were caught continuously. Only birds that had finished their post‐juvenile or post‐breeding molt, and hence were in a migratory state, were selected (Jenni and Winkler 2020). Birds caught during the species‐specific migratory period do not remain at the study site (virtually no retraps during subsequent days), hence are all in active migration.

For each bird, the visible subcutaneous fat deposit in the tracheal pit and on the abdomen was scored (scores 1–6, corresponding to scores 0–5 in Kaiser 1993). The birds were ringed, weighed to 0.1 g, the length of their third‐outermost primary was measured (Jenni and Winkler 1989), and they were thereafter released, partly after being kept in a bag for some hours (see below).

The migration strategy of each species was described by two characteristics (Table S1). (a) Migration distance: we distinguished between long‐distance migrants with their main non‐breeding grounds in sub‐Saharan Africa and short‐distance migrants having their main non‐breeding grounds in Europe south of Switzerland and in the Mediterranean basin. We considered this as part of the general migration strategy. (b) Time of day of migration over continental Europe: we distinguished between night‐migrants, that is, species migrating during the night, and day‐migrants, that is, migrating during the day. Because species may change the time of day of migration along the route (e.g., when crossing sea or desert), we considered this as part of the immediate migration strategy. Among the species examined, there were only a few which may also migrate during the other time of day at our study site: some tree pipits *Anthus trivialis* and western yellow wagtails *Motacilla flava* , usually day‐migrants at Col de Bretolet, may migrate during the night. We blood‐sampled three tree pipits (no western yellow wagtails) caught at night, which were excluded from the main analysis (results presented in Figure S3). Some starlings *Sturnus vulgaris* , usually migrating at night at Col de Bretolet, may migrate during the day, but none were blood‐sampled during the day. We excluded four Eurasian skylarks *Alauda arvensis* , a species migrating day and night, but presented their data in Figure S3.

We distinguished between three broad diet categories: frugivores, insectivores, and granivores. However, one has to keep in mind that (i) most species feed mainly, but not exclusively, on one of these food types (e.g., granivorous and insectivorous birds may also take berries, berry‐feeders also take regularly some insects) and (ii) the composition of the macro‐nutrients differs widely not only between but also within each food category. Fruits mainly contain simple sugars (mean dry mass of non‐structural carbohydrates 67%; Herrera 1987), but they may also contain a medium to high percentage of lipids (mean 6.9%, range 0.2%–58.8%; Herrera 1987; Debussche et al. 1987). Insects usually contain a high percentage of protein (mean of dry matter: 51.6%; Meyer‐Rochow et al. 2021) but some species contain a substantial part of lipids (mean of dry matter about 16%–24%; Bell 1990; Meyer‐Rochow et al. 2021) or—as aphids—sugar (Bibby and Green 1981). Grains contain predominantly starch (66%–77% polysaccharides) and usually little lipids (2%–7%) and protein (10%–40%) (Jonnalagadda et al. 2011; Khalid et al. 2023; Šramková et al. 2009).

### 2.2 Blood Sampling and Physiological State

In total, we blood‐sampled 1444 individuals of 30 passerine species (see Table S1). From 876 birds, blood was obtained within 1–15 min (mean and median 7.0, SD 3.5) after the bird flew into the mist net by puncturing the alar vein and collecting with a capillary system (Microvette C8300 Fluore, Sarstedt). These birds were used to characterize the metabolite profile during active migratory flight (in‐flight).

568 birds were left in a cloth bag for 1–13 h (mean 3.5 h, median 2.5 h, SD 3.1) before blood‐sampling (post‐flight). The long times of birds kept in bags were individuals caught in the evening who could not be released during the night but only in the early morning. The birds in the bags were usually inactive or moved only little. Hence, they were in a physiological state of inactive fasting (Jenni‐Eiermann and Jenni 1991; Jenni‐Eiermann and Jenni 2001).

### 2.3 Metabolite Determinations

We determined plasma concentrations of the three indicators of lipid catabolism: FFA, GLYC, and TG. We measured the ketone body HBA, which replaces glucose during fasting. UA, the end‐product of nitrogen metabolism, was used as an indicator of protein catabolism, and GLU as an indicator of carbohydrate metabolism.

Blood was centrifuged within 30 min, and the plasma was stored in liquid nitrogen in the field and later at −20°C until analysis after each season. The measurement of plasma concentrations of the metabolites FFA, GLYC, TG, HBA, GLU, and UA followed standard enzymatic test combinations as described earlier (Jenni‐Eiermann and Jenni 1991, 1992). Because the amounts of collected blood varied, not all metabolites could be determined in all individuals.

### 2.4 Data Analysis

In in‐flight birds, metabolite concentrations changed with the time lapse between capture and blood sampling of 1 to 15 min (Jenni‐Eiermann and Jenni 2001). In order to reflect the metabolic profile of flying birds, while avoiding extrapolating to values not well supported by the data (e.g., time lapse 0), we corrected the metabolite concentrations to a time lapse between capture and blood sampling of 3 min. In post‐flight birds, we corrected changes in metabolite levels with the time the birds were held in cloth bags (1 to 13 h) to their mean of 210 min. For in‐flight and post‐flight birds, we separately fitted a linear‐quadratic regression of the metabolite value vs. time lapse, both on the log‐scale (Figure S1). The standardized value was then the original value shifted along the linear‐quadratic regression line to a time lapse of 3 min for in‐flight and 210 min for post‐flight birds.

In an earlier study, we found changes in certain metabolites over the course of the night in three species of night‐migrants, showing an initial phase after take‐off of about 1–2 h of increasing metabolism, a phase during the middle of endurance flight, and a phase of about 1.5 h before landing of decreasing metabolism (Jenni‐Eiermann et al. 2024). In three species of day‐migrants with sufficient sample sizes (tree pipit, western yellow wagtail, Eurasian chaffinch *Fringilla coelebs* ), we found no significant changes in metabolite levels over the day (unpubl. data). Probably, an initial phase after take‐off was also present in day‐migrants (as in homing pigeons; Schwilch et al. 1996), but not recognizable because take‐off is not as synchronized as in night‐migrants. In order to include all metabolic phases of night‐ and day‐migrants, we decided not to correct for the changes in metabolite levels over the course of the night in night‐migrants. There were no significant differences in mean time of day of night migrants between groups (diet × short/long‐distance migrants) which could have confounded differences in metabolite levels between these groups.

Because the metabolism might vary with the size of the bird species, we used log (mean body mass) of the individuals examined in this study as a measure of the size of a species (see Table S1). In order to separate the effects of size and of the amount of energy stores, we used the mean body mass of the species as a measure of size and the fat score of the individual as a measure of energy stores. In the small passerines investigated here, body mass varies more with the amount of energy stores than with intra‐specific variation of size.

To investigate whether migration strategies, diet, or size of species affected the plasma metabolite pattern, we assigned the following variables to each species: day‐ or night‐migrant, short‐distance or long‐distance migrant, mainly frugivorous, insectivorous, or granivorous, mean body mass per species (Table S1). For each individual, fat score and physiological stage (in‐flight or post‐flight) were assigned.

All frugivores were night‐migrants and all granivores were day‐migrants, while insectivores included day‐ and night‐migrants. Therefore, 4 groups were built, namely night‐migrating frugivores, night‐migrating insectivores, day‐migrating insectivores, and day‐migrating granivores.

In each model the respective metabolite was included as a dependent variable (log‐transformed (log(x + c/d)), with c = 25% quantile^2^ and d = 75% quantile of non‐zero values; Stahel 2000), and the variables mentioned above as predictors (log‐transformed log(x + c/d), size and fat score centred and scaled to one standard deviation). The interaction between physiological stage (in‐flight vs. post‐flight) and the 4‐level factor combining food and day/night‐migration was included in all models as we had a specific interest in this effect. Further interaction terms were included if they improved the model based on the WAIC (Watanabe‐Akaike information criterion, Gelman et al. 2014) by more than 4 points; we sequentially added interactions between physiological stage and other predictors (excluding size at this stage), then diet/day/night‐migration with other predictors (excluding size), then fat score with other predictors (excluding size), then size with other predictors. Species was included as a random factor.

To account for phylogenetic relationships, we used the R package rotl (3.1.0, Michonneau et al. 2016) to create a phylogenetic tree of our species using the tree by Hedges et al. (2015). The phylogenetic tree was then expressed as a variance–covariance matrix using the vcv.phylo function (ape package, 5.8–1, Paradis and Schliep 2019); this matrix was then included in the model fitted with brm (package brms 2.22.0, Bürkner 2017, adding (1||gr(spec_name, cov = tree_vcv)) and data2 = list(tree_vcv = phylo)).

We assumed a Gaussian data distribution and checked model assumptions using qq‐plots and residual vs. predictor plots. Models were fitted in R (version 4.3.1, R Core Team 2023) using the function lmer from package lme4 (1–1.33, Bates et al. 2015). Following the Bayesian approach, we then used sim (package arm 1.13–1, Gelman and Su 2022) to draw from the posterior distribution and calculated point estimates and 95% uncertainty intervals (median, 2.5% and 97.5% quantiles of marginal posterior distributions).

## 3 Results

**FIGURE 1:**
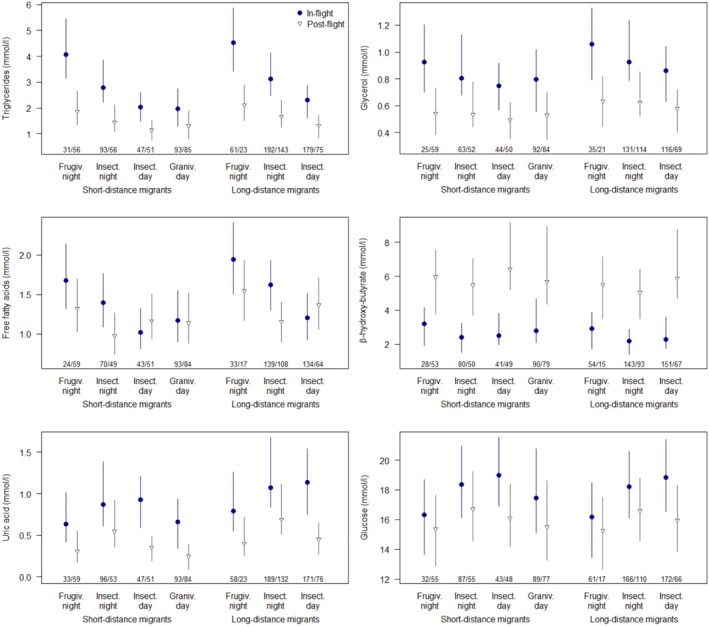
Mean metabolite concentrations (with 95% uncertainty interval) for birds caught out of active migratory flight (in‐flight, blue dots) and resting while fasting (post‐flight, gray open triangles) of four groups of main diet (frugivores, insectivores, granivores) combined with migratory behavior (nocturnal or diurnal migration) for short‐distance migrants (left) and long‐distance migrants (right, there were no granivorous long‐distance migrants). The estimates are derived from the models of Table 1 for mean size of species and mean fat score. Numbers indicate sample sizes for in‐flight and post‐flight birds.

Birds during active migratory flight had different metabolite levels than post‐flight (Figure 1). Plasma levels of TG, GLYC, UA, and GLU were higher in‐flight than post‐flight (with rather large uncertainty for GLU, but even for frugivorous night migrants the credible interval for the difference did marginally not contain 0, Table 1). For FFA, this was only the case for night‐migrants, while day‐migrants had similar levels in‐flight and post‐flight. In contrast, post‐flight HBA concentrations were about twice as high as in‐flight.

**TABLE 1:** Model estimates per predictor (rows) for each metabolite (columns; each column corresponds to a fitted model). Metabolites were log‐transformed, size log‐ and *z*‐transformed (centred and scaled to one standard deviation), and fat score *z*‐transformed.

	Triglycerides (TG)	Glycerol (GLYC)	Free fatty acids (FFA)	ß‐hydroxy‐butyrate (HBA)	Uric acid (UA)	Glucose (GLU)
Intercept	**0.96** (0.79; 1.12)	**−0.18** (−0.33; −0.03)	**0.60** (0.45; 0.74)	**1.92** (1.68; 2.14)	**−0.73** (−0.99; −0.44)	**3.30** (3.24; 3.37)
Size of species	−0.01 (−0.06; 0.03)	**−0.06** (−0.11; −0.02)	**−0.05** (−0.09; −0.00)	0.02 (−0.05; 0.09)	0.04 (−0.05; 0.12)	**−0.01** (−0.03; 0.00)
Insect.‐night^a^	−0.18 (−0.37; 0.03)	−0.01 (−0.18; 0.17)	**−0.21** (−0.38; −0.03)	−0.07 (−0.35; 0.21)	**0.41** (0.05; 0.73)	0.05 (−0.03; 0.12)
Insect.‐day^a^	**−0.32** (−0.53; −0.12)	−0.06 (−0.25; 0.12)	−0.09 (−0.27; 0.09)	0.06 (−0.22; 0.37)	0.10 (−0.30; 0.42)	0.03 (−0.06; 0.10)
Graniv.‐day^a^	**−0.24** (−0.47; −0.01)	−0.02 (−0.22; 0.17)	−0.11 (−0.28; 0.09)	−0.05 (−0.36; 0.29)	−0.12 (−0.54; 0.24)	0.01 (−0.09; 0.09)
Long‐dist^b^	**0.09** (0.00; 0.19)	**0.10** (0.02; 0.18)	**0.12** (0.02; 0.21)	−0.07 (−0.21; 0.06)	0.17 (−0.02; 0.37)	−0.01 (−0.04; 0.04)
Fat score	**0.04** (0.02; 0.05)	**0.02** (0.00; 0.03)	**−0.03** (−0.05; −0.01)	−0.01 (−0.03; 0.01)	**−0.07** (−0.10; −0.03)	**0.01** (0.00; 0.01)
In‐flight^c^	**0.61** (0.54; 0.69)	**0.38** (0.28; 0.47)	**0.18** (0.07; 0.29)	**−0.52** (−0.65; −0.38)	**0.52** (0.39; 0.65)	**0.03** (0.00; 0.07)
Insect.‐night × in‐flight	**−0.13** (−0.22; −0.05)	−0.09 (−0.21; 0.02)	0.07 (−0.06; 0.20)	−0.15 (−0.31; 0.01)	**−0.15** (−0.30; 0.00)	0.02 (−0.02; 0.06)
Insect.‐day × in‐flight	**−0.22** (−0.32; −0.13)	−0.10 (−0.21; 0.02)	**−0.27** (−0.39; −0.14)	**−0.25** (−0.41; −0.10)	**0.21** (0.05; 0.38)	**0.06** (0.02; 0.10)
Graniv.‐day × in‐flight	**−0.33** (−0.44; −0.23)	−0.10 (−0.22; 0.03)	**−0.16** (−0.30; −0.02)	−0.07 (−0.24; 0.10)	0.16 (−0.02; 0.34)	0.03 (−0.01; 0.08)
Fat score × in‐flight	—	—	—	—	**0.07** (0.02; 0.12)	—
Size × in‐flight	—	**0.07** (0.03; 0.11)	—	**−0.09** (−0.14; −0.04)	—	—

*Note:* Where the 95% uncertainty interval (in parentheses) does not include zero, the point estimate is given in bold. Only interaction terms that improved the model based on the WAIC (Watanabe‐Akaike information criterion) were included.

^a^ Baseline level: Frugivore‐night.

^b^ Baseline level: Short‐distance.

^c^ Baseline level: Post‐Flight.

The four groups combining day/night‐migration and diet differed strongest for TG and FFA, and least for GLYC and HBA (Figure 1 and Table 1). During endurance flight, frugivores showed the highest plasma concentrations of TG and FFA, followed by night‐migrating insectivores and both groups of day‐migrants. UA concentrations were higher in insectivores than in frugivores or granivores. GLU showed a similar pattern, but with more uncertainty. Plasma concentrations of GLYC and HBA differed less between the groups. Hence, the metabolic pattern is affected by a combination of diet and daytime of migration. While night‐migrants showed the highest and day‐migrants the lowest indicators of lipolysis (TG, FFA), insect feeders showed the highest indicators of proteolysis and gluconeogenesis (UA, GLU).

Long‐distance migrants had statistically well‐supported higher TG, GLYC, and FFA than short‐distance migrants (Table 1). UA showed a similar pattern but was statistically less well‐supported. GLU and HBA levels did not differ between the two categories. The effect of long/short‐distance migration on metabolites was additive and did not show strong interactions with day/night‐migration and diet.

**FIGURE 2:**
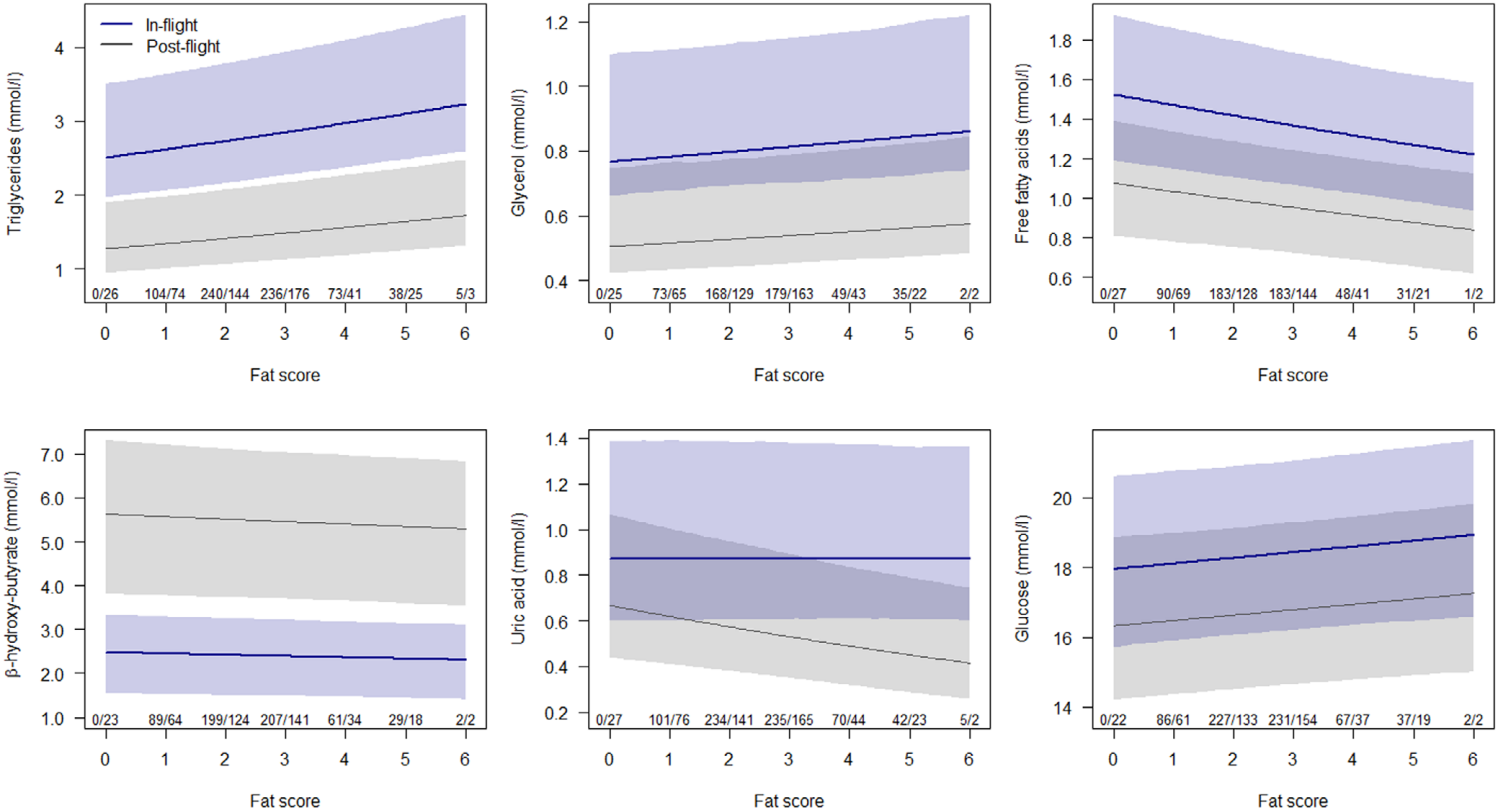
Relationship (with 95% uncertainty interval) of metabolite concentrations with fat score for birds caught out of active migratory flight (in‐flight, blue line and shade) and resting while fasting (post‐flight, gray line and shade). The estimates were derived from the model of Table 1 for short‐distance night‐migrating insectivores of mean size of species. Numbers indicate sample sizes for in‐flight and post‐flight birds.

Fat stores were positively correlated with TG, GLYC, and GLU levels, and negatively with FFA and, in post‐flight birds, with UA levels, while there was no clear correlation with HBA (Table 1 and Figure 2).

Size of species was negatively correlated with FFA and GLU levels, as well as in flying birds with HBA, and in post‐flight birds with GLYC levels (Table 1 and Figure S2).

## 4 Discussion

In this study we compared the metabolic profile among 30 passerine species during active migratory flight over an Alpine pass and post‐flight. In comparison to the flying birds, the post‐flight birds showed lower metabolite levels except HBA and, in day‐migrants, FFA. During flight TG and FFA levels were affected by diet (high in frugivores), immediate migration strategy (high in night‐migrants) and general migration strategy (high in long‐distance migrants). In contrast, UA and GLU levels were mainly affected by diet (high in insectivores), while migration strategies had a minor effect. HBA and GLYC were not noticeably dependent on day/night‐migration or diet.

Body size affects metabolic rate; the larger the organism, the lower the metabolic rate and the density of mitochondria in organs (Schmidt‐Nielsen 1984; Savage et al. 2007). We therefore expected a negative relationship between body size and plasma metabolite concentrations involved in energy metabolism. Although the range of body size was restricted (range 8–23 g plus only three species of 70, 81 and 85 g), this was confirmed for FFA, GLU, GLYC (post‐flight), and HBA (in‐flight), but not for TG and UA (Figure S2). The effect of body size was accounted for in the analyses.

### 4.1 Effect of Migratory Flight: In‐Flight Versus Post‐Flight

The post‐flight birds were usually inactive or moved only a little in the cloth bag. Hence, they were in a physiological state of inactive fasting, although their metabolism might be higher than normal because of the stress due to capture. Therefore, the differences in metabolite levels between non‐stressed inactive fasting birds and actively migrating birds might be larger than shown here.

As expected from earlier studies, all species relied on energy derived from fat and protein during flight (Jenni‐Eiermann and Jenni 1991; Jenni‐Eiermann and Jenni 2001; Rothe et al. 1987; Lindström and Piersma 1993; Schwilch et al. 2002); however, to a variable extent depending on diet type and migration strategy (see below). The indicators for fat catabolism TG, GLYC, and FFA, and the indicator for proteolysis UA are increased in‐flight compared to post‐flight. In day‐migrants, FFA values were about the same post‐flight and in‐flight. The latter might be explained by the continued lipolysis which supplies the post‐flight birds with energy and precursors for ketogenesis (Jenni‐Eiermann and Jenni 2001). Contrary to the high levels in night‐migrants, lipolysis in day‐migrants is on a moderate level and therefore FFA levels are probably not reduced any further post‐flight and might therefore even slightly increase due to the reduced energy demand.

Ketone bodies were significantly lower in‐flight than post‐flight independent of diet and migration strategy. In the fasting state, HBA replaces GLU and reduces lipolysis and proteolysis (Féry and Balasse 1983; Newman and Verdin 2014). At the same time, HBA suppresses gluconeogenesis and muscle glycolysis, thereby inducing replenishment of muscle glycogen stores (Féry and Balasse 1983). These effects explain why the in‐flight HBA levels are not at maximum but at intermediate levels, so that they do not inhibit lipolysis and proteolysis, but are high post‐flight to replenish glycogen (Jenni‐Eiermann and Jenni 2001).

Correspondingly, plasma GLU levels showed the opposite pattern; they were higher in‐flight than post‐flight. This result agrees with earlier studies reporting that under prolonged fasting and when glycogen stores are low, blood GLU levels are either maintained constant or slightly reduced (e.g., Cherel et al. 1988; Jenni‐Eiermann and Jenni 1991).

### 4.2 Lipolysis: A Consequence of Migration Strategy and Diet

The highest TG and FFA levels were observed in night‐migrants, indicating a high lipolysis during endurance flight. Fat—in comparison to glycogen and protein—was shown to be the optimal fuel for long‐lasting flights without rest or food intake (Jenni and Jenni‐Eiermann 1998). Night‐migrating small passerines optimize the transport of fatty acids in the blood to the flight muscles by using triglycerides packed in very low density lipoproteins (Jenni‐Eiermann and Jenni 1992; Zimin et al. 2024). Therefore, it was not surprising that they showed the highest TG and FFA levels.

The day‐migrants, independent of their diet, showed lower TG and FFA levels; hence, a comparatively lower lipolysis than night‐migrants, supporting the hypothesis that migration strategy affects fuel use. GLYC, which originates predominantly from hydrolysation of TG in adipose and other tissues (Jensen et al. 2001) showed the same pattern; however, statistically not well supported. Achten and Jeukendrup (2004) reported that the rate of appearance of GLYC does not increase in parallel with that of FFA during high‐intensity exercise. Part of GLYC is used for glyceroneogenesis to support the triglyceride‐fatty acid cycle; part of GLYC supports gluconeogenesis (de Andra Junior 2017). Hence, GLYC does not directly serve as an energy substrate such as GLU and FFA and therefore showed less distinct differences between the diet and migration strategy groups.

Because day‐migrants fly shorter distances per day, they do not need large fat deposits and hence rely less on fat as fuel than night‐migrants. In line with this result, two species of short‐distance day‐migrants, reed bunting *Emberiza schoeniclus* and great tit *Parus major* , were shown to increase their oxidative capacity (cytochrome oxidase) when migration approached, but not their fat oxidation capacity measured as the 3‐hydroxyacyl CoA‐dehydrogenase (HAD) activity (Lundgren and Kiessling 1985). Also, the anecdotal evidence from tree pipits and Eurasian skylarks, both day‐ and night‐migrating species, supported this hypothesis. The few night‐migrating individuals which we caught on our Alpine pass had higher TG and FFA levels (and less UA) than their day‐migrating conspecifics (Figure S3).

Lipid use was further influenced by diet type. The frugivorous species had by far the highest plasma TG and FFA levels (Figure 1). We assume that a fruit diet enables this high reliance on lipids during flight. Most fruits mainly contain simple sugars, and it was shown for several species that about 80% of GLU can be passively absorbed; hence little energy is required for uptake (Caviedes‐Vidal and Karasov 1996). Sugars can be converted to FFA, synthesized to TG, and finally stored as fat (Flatt 1970). Fruits may also contain a substantial part of lipids, and some species selectively feed on fat‐rich berries (Herrera 1998; Bairlein 1994). Such a high‐fat but non‐ketogenic diet induces an increase in enzyme activities involved in fatty acid synthesis but also in fat oxidizing enzymes (Newman and Verdin 2014; Levey and del Martinez Rio 2001; Lundgren 1988). At the same time, many fruits contain little protein. A diet with a high proportion of protein induces low fat deposits, while a low‐protein diet (but still sufficient to maintain a positive nitrogen balance) results in fat birds (Kirkpinar and Oguz 1995; Klasing 1998; Rosebrough and McMurtry 1993; Skrip and McWilliam 2016; Guglielmo et al. 2017). This is probably due to an intracellular competition between the acetyl‐CoA carboxylase pathway (fatty acid synthesis) and the aconitase‐isocitrate dehydrogenase pathway (citrate cycle) for limited cytoplasmic citrate, which is needed to produce α‐ketoglutarate for transamination when metabolizing protein (Rosebrough and McMurtry 1993). Indeed, frugivorous species were shown to be more likely to gain body mass during stopover than strict insectivores (Levey and del Martinez Rio 2001). In concert with an increase in body fat, fat oxidizing enzymes increase, and the oxidative capacity of the flight muscles is up‐regulated (Lundgren and Kiessling 1985; Driedzic et al. 1993; McFarlan et al. 2009; Lundgren 1988). The frugivores in our study also had the highest fat reserves of our four diet groups (mean fat score: frugivores 3.43 ± 1.12 SD, insectivores night 2.71 ± 1.05, insectivores day 2.22 ± 0.87, granivores 2.37 ± 0.89). And in‐flight lipid use, indicated by TG and GLYC levels, increased with increasing fat stores (Figure 2). Hence, frugivores appear to be best adapted to use a maximum of lipids during migration.

Night‐migrating insectivores also rely mainly on energy derived from fat during flight, but to a smaller extent. A diet with a high percentage of protein induces lower fat deposits (see above). The insectivore night‐migrants in our study had indeed lower fat stores than the frugivores (see above).

A seed diet also seems less optimal to enhance lipid metabolism. Starch must first be broken down into monosaccharides (e.g., glucose) which then can be absorbed into the blood. In yellow‐rumped warblers *Setophaga coronata* , extraction efficiency of starch was very poor despite a long retention time (Afik and Karasov 1995). Moreover, granivorous species do not show a pronounced seasonal increase in food assimilation efficiency as insectivorous and frugivorous birds do (Berthold 1996). To replenish their fuel stores, they have time to forage during the afternoon or, if they cannot fully compensate for the energy loss during the preceding flight, they might stopover for some full days (Newton 2008). However, one should keep in mind that there are also granivorous long‐distance migrating species, such as ortolan bunting *Emberiza hortulana* and turtle dove *Streptopelia turtur* , which accumulate large fat deposits to cross the Mediterranean and the Sahara (Schumm et al. 2021; Selstam et al. 2015).

HBA levels did not differ between diets or day/night‐migration, although HBA is a product of fat catabolism (Burke et al. 2000). HBA replaces GLU as the primary energy source in the CNS during fasting and does not fuel flight. Therefore, a dependence of HBA levels on diet and migration strategy was not expected.

### 4.3 Proteolysis and Glucose Levels: A Matter of Diet

The highest UA levels were found in insectivores, both day and night‐migrants, indicating a higher proteolysis during flight than in frugivores and granivores. Similarly, high plasma UA levels were found during spring migration in several species of insectivorous migrants, while plasma UA levels of frugivorous and nectarivorous species were much lower (Gannes et al. 2001; Jenni‐Eiermann and Jenni 2003).

Actively flying migrants are in a fasting state and therefore do not digest dietary protein. The elevated UA levels are thus caused by an increased protein metabolism due to a protein‐rich diet and oxidation of endogenous protein. The catabolism of endogenous protein is necessary to provide the fasting and exercising metabolism with glucogenic amino acids and may maintain water balance under ambient conditions of dehydration, but quantitatively plays a minor role as energy fuel (Jenni and Jenni‐Eiermann 1998). However, the increased UA levels in insectivores suggest that they derive more energy from protein oxidation than frugivores and granivores. Also, the increased proteolysis delivers gluconeogenic amino acids that are probably responsible for the high glucose levels of the insectivores.

The granivores, all day‐migrating, showed about the same low UA levels as the night‐migrating frugivores; hence, they kept protein oxidation at a minimum. However, in contrast to frugivores, they also had low TG and FFA levels, indicating lower fat use. Their high glucose levels might indicate that they also use glycogen, in addition to fat, for their short flight bouts and intermittent flight pattern. They can refill their glycogen reserves more frequently than the night‐migrants flying longer distances. Lundgren and Kiessling (1985) showed that species which do not deposit much fat towards migration did enhance their glycolytic oxidative capacity but not their fatty acid oxidation capacity, suggesting an increased reliance on carbohydrate metabolism. However, the rate of utilization of GLU during migratory flight is unknown, and the few studies measuring the usually small glycogen reserves in migrants vary widely, probably due to technical and methodological differences (Dawson et al. 1983; Marsh 1983). Whether day‐migrating granivores with their intermittent flight mode have larger glycogen stores or are able to refill them efficiently, so that they can substantially contribute to flight energy, remains unclear. Further studies on glycogen content in in‐flight migrants of different diet types are needed to elucidate the fuel types used by day‐migrating granivores.

### 4.4 Effect of Fat Stores

TG and GLYC levels were positively correlated with fat stores in both in‐flight and post‐flight birds. From medical studies it is known that high fat stores are associated with elevated plasma concentrations of TG in the fasted state (Hardman 1999). However, this was not true for FFA levels, which were negatively correlated with fat stores. A possible explanation might be that fat birds resynthesise FFA into TG to a higher extent than lean birds.

HBA levels did not vary with fat stores. HBA replaces GLU in the CNS during fasting. Since the energetic need of the CNS does not depend on fat stores, a correlation between HBA and fat stores was not expected.

UA levels decreased with fat score in post‐flight birds. This is in line with earlier results showing that inactive fasting birds, which dispose of high initial fat deposits, reduce the percentage of energy derived from protein (Jenni and Jenni‐Eiermann 1998). The question of why in‐flight birds did not show the same relationship remains unclear.

Interestingly, in fat birds, more plasma GLU was present, both in‐flight and post‐flight. This result agrees with Lundgren and Kiessling (1986) who observed an increased activity of 6‐phosphofructokinase (PFK), a key enzyme of glycolysis, in migratory birds with high fat deposits. They explained this on first view paradoxical result with an increased glycolytic rate during periods of high substrate turnover, as during heavy exercise, even if fat is the major substrate.

### 4.5 Effect of Long‐ and Short‐Distance Migration Strategy

In contrast to day/night‐migration, which is part of the immediate migration strategy when crossing the Alps, long‐ and short‐distance migration is part of the general migration strategy, as the Alps are situated well before the two ecological barriers, the Mediterranean Sea and the Sahara, on autumn migration. In contrast to short‐distance migrants, long‐distance migrants must cover large distances with no or limited opportunities to land and refuel. It appears that the metabolic adaptations to cross these inhospitable areas are already present when flying over continental Europe. Long‐distance migrants had significantly increased levels of TG, FFA, and GLYC, which indicate an increased lipid catabolism in‐flight and post‐flight.

In our sample, long‐ and short‐distance migrants were present in all diet categories and among day‐and night‐migrants (only granivores were all short‐distance day‐migrants). Moreover, we did not find any significant interaction of short/long‐distance migration with other factors. Therefore, the up‐regulation of lipolysis in long‐distance migrants is additive. Lundgren (1988) found in Sweden that long‐distance migrants preparing for autumn migration have high levels of oxidative enzymes showing an increased aerobic capacity, including the fat‐oxidizing capacity (HAD) compared to short‐distance migrants, which increased their oxidative capacity but not their HAD. Hence, long‐distance migrants are better prepared for endurance flight than short‐distance migrants, and they start to prepare before or at the beginning of migration, although most species accumulate extensive fuel loads only near the ecological barriers (Alerstam and Lindström 1990; Fransson et al. 2008; Schaub and Jenni 2000a, 2000b), well after they have crossed the Alps.

## 5 Conclusion

This study showed that migrant passerines vary in the degree of fat use, depending on migration strategy, diet, and current fat stores. Although fat is still the main fuel, day‐migrating short‐distance migrant granivores and insectivores seem to use additional fuel such as glycogen (granivores) or protein (insectivores) to a higher degree than other species. The short flight bouts apparently do not require a substantial up‐regulation of fatty acid transport and oxidation. The longer flight bouts of night migration seem to require a higher degree of fat use. Species switching from a protein‐rich arthropod diet to a low‐protein diet consisting mainly of berries before migration avoid the competition between protein deamination and fat deposition and hence can maximize fat deposition and use during endurance flight even further. Additionally, long‐distance migrants favor fat use even before crossing the inhospitable Mediterranean Sea and the Sahara. Hence, long‐distance night‐migrant frugivores with high fat loads appear best adapted for fat use during migratory flight.

This study shows that not all migrants maximize fat use for migration, only those that cover large bouts non‐stop. Therefore, the metabolic requirements and adaptations for maximum fat use appear to be a costly affair that is avoided for short flights.

## Author Contributions

**Susanne Jenni‐Eiermann:** conceptualization (equal), data curation (equal), formal analysis (equal), methodology (equal), project administration (equal), writing – original draft (equal), writing – review and editing (equal). **Lukas Jenni:** conceptualization (equal), data curation (equal), formal analysis (equal), writing – original draft (equal), writing – review and editing (equal).

## Conflicts of Interest

The authors declare no conflicts of interest.

## Statement on Inclusion

Field work included local students and volunteers. Literature published by a scientist from the region was cited.

## Supporting information


Data S1.

## Data Availability

Data of the metabolites are available at: S. Jenni‐Eiermann and L. Jenni: Plasma metabolites of 30 bird species during active migratory endurance flight. vogelwarte.ch Open Repository and Archive: https://doi.org/10.5281/zenodo.15083572.
