# Supplementary material for: Migration Strategy and Diet Affect the Metabolism of Passerine Birds During Endurance Flight
Source: Ecol Evol. 2025 Apr 15;15(4):e71299. doi: 10.1002/ece3.71299 (PMC12000537; doi:10.1002/ece3.71299)

# Migration strategy and diet affect the metabolism of passerine birds during endurance flight

**Supporting Information**

## Table S1

Species with their characteristics and sample sizes. Day- or night-migrant according to Winkler (1999: Avifauna der Schweiz. Der Ornithologische Beobachter, Beiheft 10: 1–252), Short/long-distance migrant and main diet according to the handbooks by Cramp *et al.* (1988 – 1994: Handbook of the Birds of Europe, the Middle East and North Africa. The Birds of the Western Palearctic, Vol. 5-9. Oxford University Press, Oxford) and Glutz *et al*. (1985-1997: Handbuch der Vögel Mitteleuropas, Vol. 10-14. Akademische Verlagsgesellschaft; Aula, Wiesbaden). Size of species is the mean body mass of the individuals examined in this study. Because the amounts of collected blood varied, not all metabolites could be determined in all individuals. Therefore, the sample sizes of specific metabolites may be smaller than given here (see Figures 1 and 2).

| **Species** |  | **Day or night migrant** | **Long- or short-distance migrant** | **Primary diet** | **Size of species (g)** | **Sample size post-flight** | **Sample size in-flight** |
| --- | --- | --- | --- | --- | --- | --- | --- |
| Acrocephalus scirpaceus | Common Reed Warbler | night | L | insects | 13.6 | 15 | 12 |
| Anthus pratensis | Meadow Pipit | day | S | insects | 16.8 | 14 | 7 |
| Anthus trivialis | Tree Pipit | day | L | insects | 21.3 | 42 | 95 |
| Carduelis citrinella | Citril Finch | day | S | seeds | 13.0 | 12 | 4 |
| Curruca communis | Common Whitethroat | night | L | fruit | 15.7 | 1 | 4 |
| Cyanistes caeruleus | Eurasian Blue Tit | day | S | insects | 10.4 | 14 | 12 |
| Erithacus rubecula | European Robin | night | S | insects | 16.4 | 87 | 141 |
| Ficedula hypoleuca | European Pied Flycatcher | night | L | insects | 12.8 | 75 | 175 |
| Fringilla coelebs | Eurasian Chaffinch | day | S | seeds | 21.0 | 24 | 57 |
| Fringilla montifringilla | Brambling | day | S | seeds | 22.5 | 9 | 6 |
| Hippolais icterina | Icterine Warbler | night | L | insects | 13.9 | 2 | 5 |
| Linaria cannabina | Common Linnet | day | S | seeds | 19.0 | 6 | 5 |
| Locustella naevia | Common Grasshopper W. | night | L | insects | 15.5 | 1 | 3 |
| Luscinia megarhynchos | Common Nightingale | night | L | insects | 23.1 | 1 | 3 |
| Motacilla alba | White Wagtail | day | S | insects | 19.1 | 1 | 2 |
| Motacilla cinerea | Grey Wagtail | day | S | insects | 17.1 | 0 | 3 |
| Motacilla flava | Western Yellow Wagtail | day | L | insects | 15.7 | 43 | 111 |
| Muscicapa striata | Spotted Flycatcher | night | L | insects | 15.3 | 9 | 13 |
| Oenante oenanthe | Northern Wheatear | night | L | insects | 23.4 | 27 | 17 |
| Phoenicurus phoenicurus | Common Redstart | night | L | insects | 14.5 | 28 | 17 |
| Phylloscopus trochilus | Willow Warbler | night | L | insects | 8.1 | 5 | 6 |
| Prunella modularis | Dunnock | day | S | insects | 18.0 | 23 | 27 |
| Saxicola rubetra | Whinchat | night | L | insects | 16.3 | 9 | 7 |
| Serinus serinus | European Serin | day | S | seeds | 11.7 | 14 | 15 |
| Spinus spinus | Eurasian Siskin | day | S | seeds | 12.5 | 20 | 18 |
| Sturnus vulgaris | Common Starling | night | S | fruit | 84.7 | 11 | 10 |
| Sylvia atricapilla | Eurasian Blackcap | night | S | fruit | 19.0 | 25 | 13 |
| Sylvia borin | Garden Warbler | night | L | fruit | 19.4 | 27 | 75 |
| Turdus merula | Common Blackbird | night | S | fruit | 80.5 | 3 | 2 |
| Turdus philomelos | Song Thrush | night | S | fruit | 70.4 | 20 | 11 |

## Figure S1

Dependence of metabolite concentrations on time lapse between capture and blood sampling. Data of in-flight birds are in black, post-flight birds in orange, both with their corresponding linear-quadratic regression lines and 95% uncertainty intervals (dotted lines). We corrected the original plasma concentrations to a time lapse of 3 min for in-flight and 210 min for post-flight birds (vertical dotted lines) by shifting them parallel to the corresponding regression line.


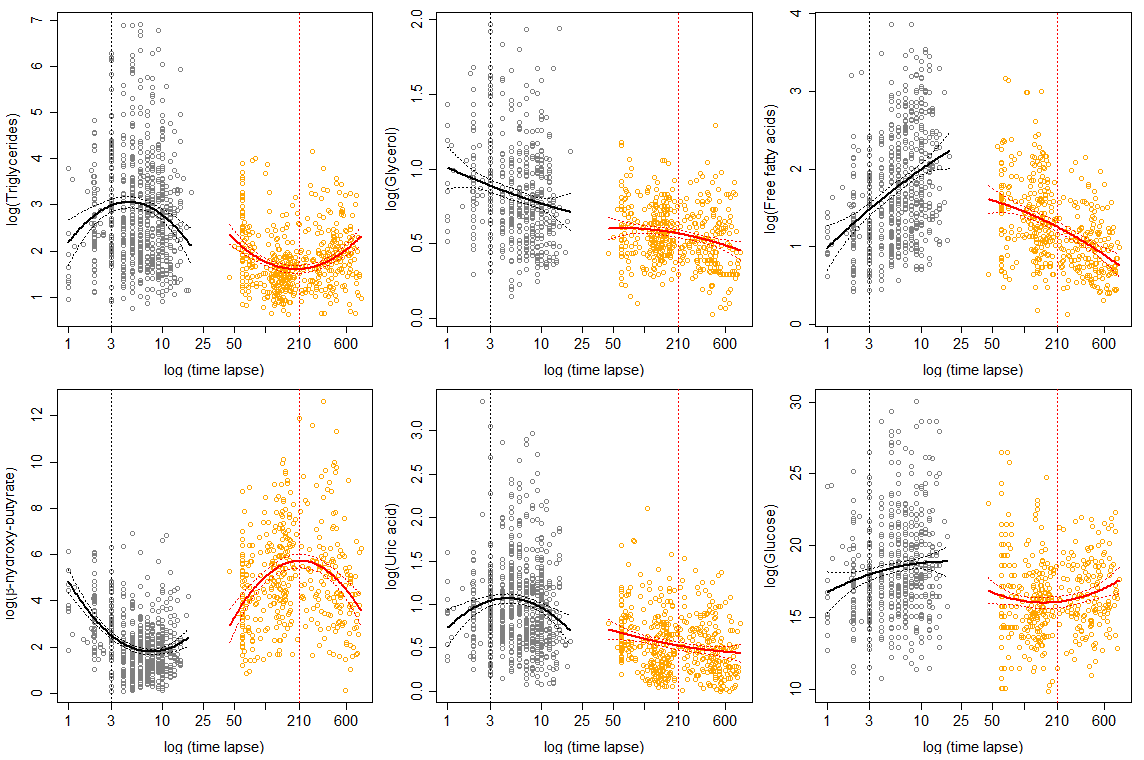


## Figure S2

Effect of body size on metabolite levels. Relationship of metabolite concentrations (with 95% uncertainty interval) with species-specific body size for birds caught out of active migratory flight (in-flight, blue line and shade) and resting while fasting (post-flight, grey line and shade). The estimates are derived from the model of Table 1 for short-distance night-migrating insectivores with a mean fat score. The available data is shown as a data rug.


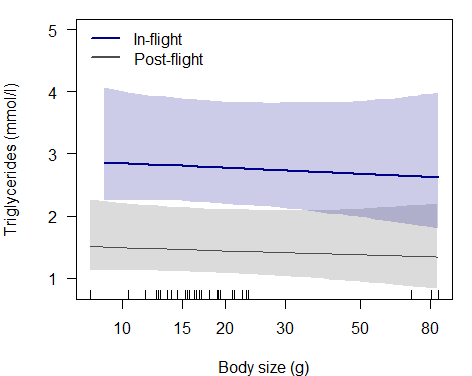

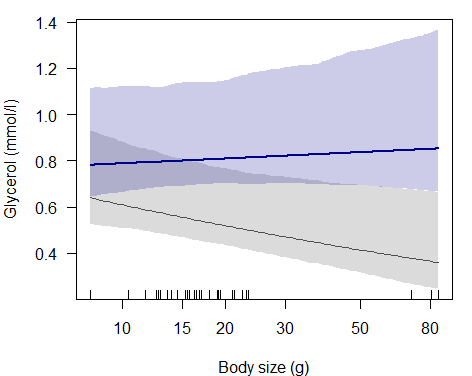

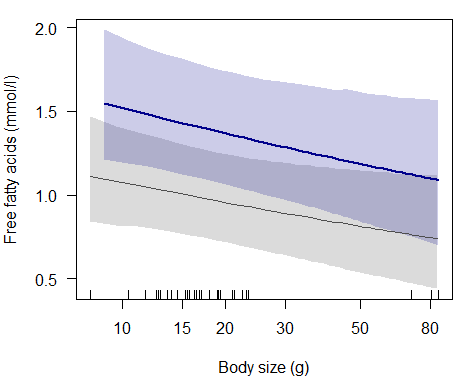

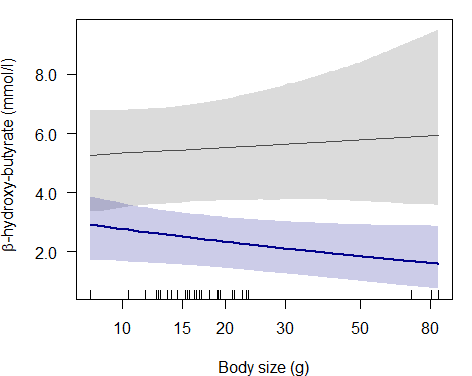

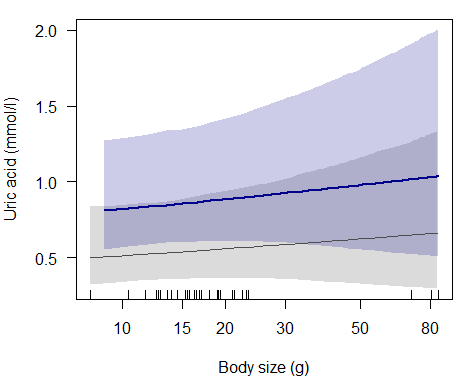

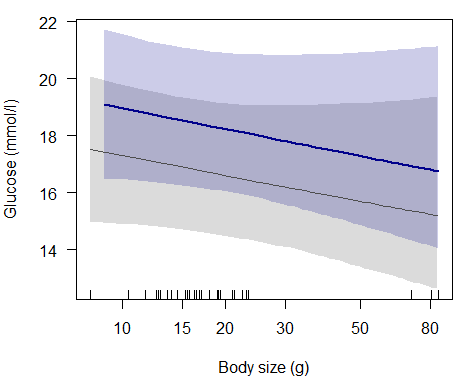


## Figure S3

Metabolite levels in day- and night-migrating tree pipits *Anthus trivialis* and Eurasian skylarks *Alauda arvensis*. Plasma metabolite concentrations (mmol/l) of three night-migrating tree pipits, one day-migrating and three night-migrating Eurasian skylarks and the mean (± 1 SD) of day-migrating tree pipits (with sample size). The intra-specific differences between day- and night-migrants in metabolite levels shown here generally agree with the inter-specific differences (Figure 1).


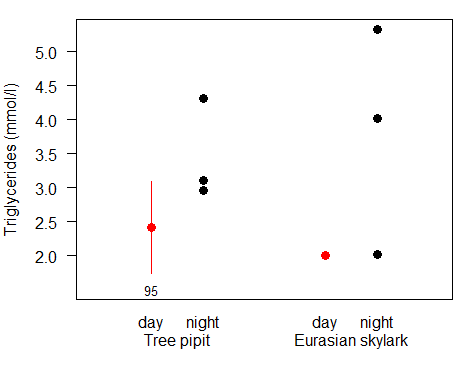

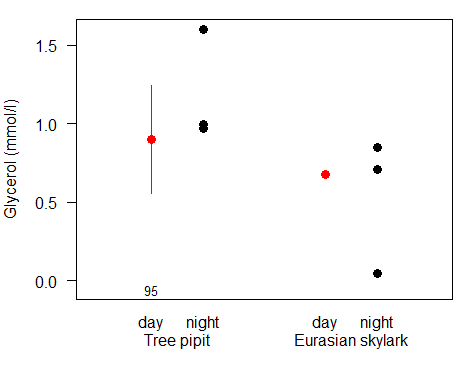


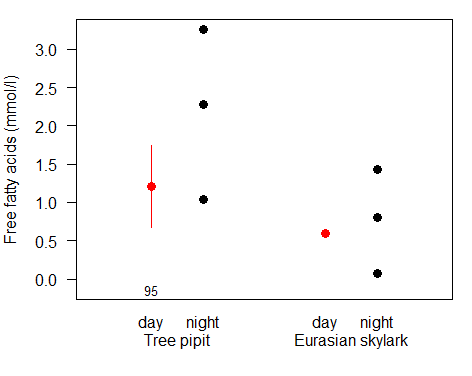

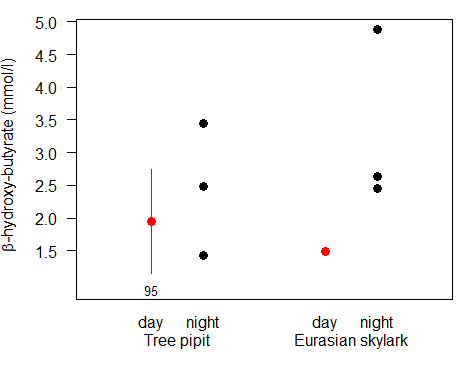


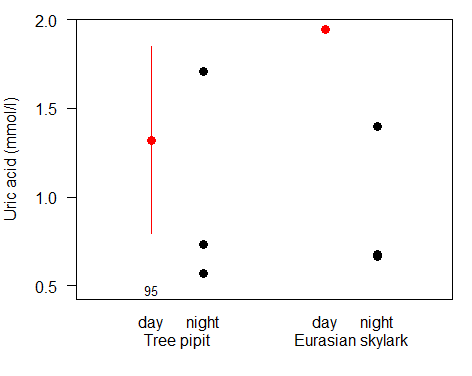

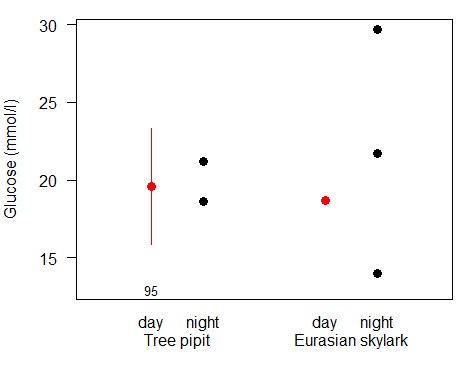

Supplement: Supplementary file 1 — Data S1. [file ECE3-15-e71299-s001.docx]
